# Cut-Point for Satisfactory Adherence of the Dietary Sodium Restriction Questionnaire for Patients with Heart Failure

**DOI:** 10.5935/abc.20190011

**Published:** 2019-02

**Authors:** Karina Sanches Machado d’Almeida, Sofia Louise Santin Barilli, Gabriela Corrêa Souza, Eneida Rejane Rabelo-Silva

**Affiliations:** 1 Programa de pós-graduação em Cardiologia e Ciências Cardiovasculares da Faculdade de Medicina da Universidade Federal do Rio Grande do Sul, Porto Alegre, RS - Brazil; 2 Clínica de Insuficiência Cardíaca do Hospital de Clínicas de Porto Alegre, Porto Alegre, RS - Brazil; 3 Programa de pós-graduação da Escola de Enfermagem da Universidade Federal do Rio Grande do Sul, Porto Alegre, RS - Brazil; 4 Departamento de Medicina Interna da Faculdade de Medicina da Universidade Federal do Rio Grande do Sul, Porto Alegre, RS - Brazil; 5 Curso de Nutrição da Universidade Federal do Pampa, Itaqui, RS - Brazil

**Keywords:** Heart Failure/physiopathology, Sodium,Dietary, Surveys and Questionnaires, Behavior Control, Decision Making, Muklticenter Study

## Abstract

**Background:**

The low or non-adherence to reduction of sodium intake has been identified as one of the main precipitating factors of heart failure (HF). The Dietary Sodium Restriction Questionnaire (DSRQ) identifies factors that can interfere with adherence to this recommendation. However, there is still no cut-point to define adherence for this questionnaire.

**Objectives:**

To identify the cut-point for satisfactory adherence to the Brazilian version of the DSRQ, (the Questionário de Restrição de Sódio na Dieta, QRSD).

**Methods:**

Multicenter study. Patients with HF in outpatient treatment (compensated) and those treated in emergency departments due to acute HF (decompensated) were included. For the cut-point definition, the DSRQ scores were compared between groups. A ROC curve was constructed for each subscale to determine the best point of sensitivity and specificity regarding adherence. A 5% significance level was adopted.

**Results:**

A total of 206 compensated patients and 225 decompensated were included. Compensated patients exhibited scores that showed higher adhesion in all subscales (all p <0.05). Scores ≥ 40 points of a total of 45 for the subscale of Attitude and Subjective Norm; scores ≤ eight of a total of 20 for Perceived Behavioral Control; and ≤ three of a total of 15 for Dependent Behavior Control were indicative of satisfactory adherence.

**Conclusions:**

Based on the evaluation of patients in these two scenarios, it was possible to determine the cut-point for satisfactory adherence to the reduction of sodium in the diet of patients with HF. Countries with similar culture could use this cut-point, as other researchers could also use the results as a reference for further studies.

## Introduction

Reduction of sodium intake is usually part of the non-pharmacological treatment for patients with heart failure (HF), since the excessive consumption is associated with fluid retention and congestive situations.^1,2^ A poor or non-adherence to this recommendation has been identified among the main precipitating factors of HF decompensation^3-5^ and has been linked to the need for hospitalization and worse outcomes.^6,7^

To understand what factors could potentially interfere with the adherence to the reduction of sodium intake, researchers from the United States of America developed the Dietary Sodium Restriction Questionnaire (DSRQ).^8^ This instrument is based on the Planned Behavior Theory and considers three constructs: attitude, subjective norm and perceived behavioral control. Recently, the DSRQ was adapted (transculturally) and validated for the Portuguese language in Brazil, with the name *Questionário de Restrição de Sódio na Dieta* (QRSD).^9,10^ Although the DSRQ has already been the object of other studies,^11-13^ there is still no cut-point to define satisfactory adherence for the interviewed patients. Seeking to fill this gap, this study was designed to identify a cut-point for satisfactory adherence to sodium restriction when using the QRSD, both for stable patients on outpatient care, and for decompensated patients.

## Methods

### Design and sample

This is a case-control study, conducted in two institutions in southern Brazil from March 2010 to October 2014.

Adult patients, with a diagnosis of HF - reduced or preserved left ventricular ejection fraction (LVEF)^9^ - were included. Patients in outpatient treatment (compensated) and those admitted to emergency rooms due to acute HF (decompensated) participated in this study. It was used a convenience sample, with a total of 431 HF patients (206 compensated and 225 decompensated).

Patients with cognitive impairment or barrier (e.g., decreased hearing acuity, neurological sequelae) were excluded since these impairments could make it difficult for patients to fill out the questionnaire.

### Data collection

Clinical and sociodemographic data were collected from medical records. The QRSDs were administered by the researchers in a private room, with a mean duration of 40 minutes.

The Brazilian version of the DSRQ comprises 27 items, 11 descriptive questions and 16 questions divided into three subscales, which are scored using the 5-point Likert scale:^10^

- Attitude and subjective norm (nine items, with scores ranging from nine to 45) - assesses the patient’s beliefs regarding the results of performing a diet with reduced sodium and the importance of other people’s approval or disapproval of this practice;
- Perceived behavioral control (four items with scores ranging from four to 20) - assesses the patient’s ability to identify facilitators and barriers related to the reduction of sodium in their diet;
- Dependent behavior (three items with scores ranging from three to 15) - assesses the presence or absence of resources and constraints for a patient to follow a sodium-reduced diet.

In the first subscale - attitude and subjective norm - the lowest score indicates a “strong disagreement” and the highest, a “strong agreement”. In the second and third subscales - perceived behavioral control and dependent behavior - the minimum score indicates “not at all”, while the maximum indicates “a lot”.^8^

This study was approved by the Ethics Committee of the institutions involved and all participants signed a written informed consent form before taking part in this study.

### Data analysis

Data were analyzed using the Statistical Package for Social Sciences version 18.0. Continuous variables with normal distribution were expressed as mean and standard deviation and without normal distribution, as median and interquartile range. Categorical variables were expressed as absolute numbers and relative frequency. To compare continuous variables, unpaired Student’s t-test or Mann-Whitney test were used, according to data distribution. Associations between categorical variables were analyzed using the chi-square test or Fisher's exact test. A 5% significance level was adopted.

To define the cut-points, the QRSD scores were compared between compensated and decompensated patients. A ROC curve was constructed for each subscale, and an additional comparison of patients by functional class (I - II) and (III - IV) was performed to determine the best point of sensitivity and specificity regarding adherence to the diet. determine the best point of sensitivity and specificity regarding adherence to the diet.

## Results

A total of 431 HF patients participate in the study. Of the total, 206 were in outpatient treatment (compensated) and 225 patients sought emergency care (decompensated). Sociodemographic and clinical characteristics of the studied population are shown in Table 1. Mean age was 63 ± 13 years, and 59.2% of the participants were male; mean LVEF was 36.8 ± 14.0%.

**Table 1:** Characteristics of the participants

Characteristics	Compensated (n = 206)	Decompensated (n = 225)	p
**Sociodemographic**			
Age (years)*	60 ± 12	66 ± 12	< 0.001
Male (%)^†^	65.0	53.8	0.023
**Ethnicity (%)^†^**			**< 0.001**
White	85.4	57.8	
Black	9.7	16.4	
Mixed-race	4.9	25.8	
**Years of study (%)^†^**			**0.083**
Until 8 years	75.7	83.0	
9 to 11 years	19.9	12.1	
12 years or more	4.4	4.9	
**Marital status (%)^†^**			**< 0.001**
Lives with a companion	69.4	49.3	
Lives alone	30.6	50.7	
**Clinical**			
LVEF (%)*	31.3 ± 9.1	42.0 ± 15.7	< 0.001
Functional class NYHA (%)^†^			< 0.001
I	42.0	1.4	
II	34.2	20.7	
III	23.3	63.1	
IV	0.5	14.9	
**Etiology (%)^†^**			**0.002**
Ischemic	33.0	43.2	
Hypertensive	18.0	10.9	
Others	49.0	45.9	
**Medications prior to admission (%)^†^**			
Beta-blockers	85.4	69.2	< 0.001
Anti-hypertensives	96.6	87.5	0.001
Diuretics	82.5	83.9	0.795

LVEF: left ventricular ejection fraction; NYHA: New York Heart Association.

* Continuous variables described as mean ± standard deviation, unpaired Student's t test;

† categorical variables expressed as %, chi-square test.

Regarding the QRSD scores, compared with decompensated patients, compensated patients had better scores, showing greater adherence in all subscales. Mean scores for compensated and decompensated groups, and for categories of functional classes are shown in Table 2.

**Table 2:** Scores of the Dietary Sodium Restriction Questionnaire subscales for compensated and decompensated patients and for categories of functional class

	Attitude and subjective norm	Perceived behavioral control	Dependent behavior
**Situation**			
Compensated	42.6 ± 4.0	8.4 ± 4.1	5.2 ± 3.0
Decompensated	38.5 ± 6.3	10.9 ± 4.2	5.5 ± 3.0
p value	< 0.001	< 0.001	0.399
**Functional class**			
I x II	41.6 ± 5.1	8.8 ± 4.4	5.0 ± 2.8
III x III	39.3 ± 6.1	10.6 ± 4.2	5.6 ± 3.2
p value	< 0.001	< 0.001	0.038

*Continuous variables described as mean ± standard deviation

**Figure 1:**
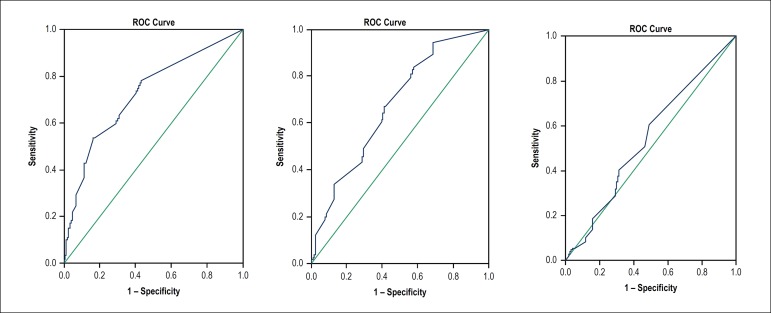
ROC curves for all three DSRQ subscales.

According to the ROC curve analysis, the area under the curve was 0.725 (95%CI; 0.677 to 0.772) for the attitude and subjective norm subscales; 0.670 (95%CI; 0.620 to 0.721) for the perceived behavioral control subscale; and 0.544 (95%CI; 0.489 to 0.598) for the dependent behavior subscale (Figure 1).

The results of the functional class analysis were 0.631 (95%CI; 0.578 to 0.685) for the attitude and subjective norm subscales; 0.628 (95%CI; 0.574 to 0.682) for the perceived behavioral control subscale; and 0.561 (95%CI; 0.506 to 0.617) for the dependent behavior subscale.

Sensitivity and specificity were, respectively, 53.8 and 83.5 for the attitude and subjective norm subscales; 68.0 and 58.3 for perceived behavioral control subscale; and 60.9 and 51.0 for dependent behavioral subscale. Cut-off points for adherence were scores greater than or equal to 40 points in the attitude and subjective norm subscale; lower than or equal to eight points for perceived behavioral control; and lower than or equal to three points for Dependent Behavior (Table 3).

**Table 3:** Cut-point for adherence established to the Dietary Sodium Restriction Questionnaire subscales

Subscales	Scores (min - max)	Cut-point (adherence)	Sensitivity (%)	Specificity (%)
Attitude and subjective norm	9 - 45	≥40	53.8	83.5
Perceived behavior control	4 - 20	≤8	68.0	58.3
Dependent behavior control	3 - 15	≤3	60.9	51.0

## Discussion

This is the first study conducted in a clinical scenario that tried to establish cut-points for the DSRQ/QRSD regarding adherence. This instrument considers the knowledge, barriers and attitudes of patients with HF regarding sodium restriction in the diet. Adherence can be defined as the degree to which individuals comply with recommendations (related to pharmacological treatment of changes in lifestyle) from the health team.^14^ In the context of HF, treatment adherence is considered an essential component to the success of self-care and prevention of complications, including hospitalizations.^15^

The sample was predominantly male patients older than 60 years, poorly educated, and with predominantly reduced LVEF, similar to other studies that addressed adherence in patients with HF.^8,16,17^

Compared with compensated patients, in decompensated patients’ group, there were fewer men, fewer people with white ethnicity and a greater number of people living alone. These characteristics have already been related to lower adherence in previous studies. Lennie et al.^11^ investigated the relationship between knowledge, attitudes, and barriers to adherence of a low-sodium diet in patients with HF, and also found similar sociodemographic characteristics, with mean age of 65 years and 32% of participants living alone. In fact, advanced age is among the main factors that contribute to high rehospitalization rates due to decompensation of HF patients.^18^ In addition to advanced age, many patients with HF have cognitive deficits, including memory loss.^19^ Regarding ethnicity, a recent study demonstrated an association between non-white race and non-adherence in patients with HF after hospital discharge.^20^ The fact of living alone can interfere with adherence, since this behavior is largely influenced by the opinion of people whom patients consider important, including spouses and family members.^12^ Lack of family support can make the patient feel alone. The inclusion of family members in the treatment of HF - mainly in relation to adherence to non-pharmacological measures - seems to be a crucial point and has been used as a strategy for self-care.^21,22^

The multifactorial causation and subjectivity related to adherence could explain the difficulty encountered by health professionals to measure patients’ commitment to a particular behavior. In this context, instruments that can provide more reliable information on patient outcomes in terms of knowledge, barriers and attitudes, with cut-points for adequate and poor adherence could help to identify factors that potentially influence this outcome.^23^

According to the researchers responsible for developing the QRSD, the instrument was built with the goal of being a self-administered tool.^8^ However, considering cultural differences between the studied populations, it is recommended that, in the Brazilian population, the QRSD be applied by means of interviews, by trained investigators. In addition, because each subscale relates to a particular construct, we sought to identify different cut-points for each of them.

High scores observed in the subscale of attitudes and subjective norm contributed to raising the cut-point (≥ 40, a total of 45 points) and indicated that patients are aware of the importance of adhering to sodium reduction, and can identify signs and symptoms associated with excessive intake, as well as benefits related to the reduction. However, as described in the literature,^7,24,25^ knowledge alone does not seem to be sufficient to ensure compliance, to which other skills are required, such as motivation and willpower.^21^ Accordingly, incorporating this measure into the routine remains a major challenge for patients.

On the same subscale, the last three questions that denote adherence are influenced by the opinion of people considered important by patients (spouse, family members, physicians and other health professionals). The inclusion of family members in the treatment of patients with HF appears to be a crucial point and is gaining more space as a strategy for self-care, with positive results in the reduction of sodium intake by these patients.^21,22^

Regarding the scores and the cut-point identified for the subscale perceived behavioral control (≤ 8, a total of 20 points), the main barriers - for both compensated and decompensated patients - are the palatability of foods with little salt, food preferences of patients, and less significantly, the willpower to change their diets, factors already described previously. Palatability of foods with low sodium content has been referred as one of the main barriers to adherence.^26,27^ Furthermore, when compared to healthy individuals, patients with HF have a preference for highly salted foods.^28^ This can be explained largely by changes in the renin-angiotensin-aldosterone system, which promotes a higher desire for salt.^29^

The low scores observed in the dependent behavior subscale influenced the determination of a low cut-point (≤ 3, of a total of 15 points). In a study conducted with a sample of 225 patients with decompensated HF,^12^ decision-making situations that occur outside the home - going to restaurants and the supermarket - did not influence significantly adherence in this population, possibly due to the limitations imposed by the severity of the disease. In addition, the trip to the supermarket and the choice of food is often performed by a family member or the person responsible for their care, which may explain the small impact caused by this factor.^6^

In the comparative analysis of patients by functional class to determine the cutoff point for satisfactory adherence, it was observed that both sensitivity and specificity values were lower than those obtained in the comparison between compensated and decompensated patients. Thus, our findings indicated that adherence was higher in outpatients compared with patients hospitalized for decompensated HF.

### Limitations

Other factors other than sodium restriction may affect HF decompensation, which can lead to a small bias in the determination of the cut-point.

Although it was a case-control study, matching was not sufficient to minimize discrepancies between the two groups (compensated and decompensated). Other studies with the same design may contribute to elucidate the findings of this study.

Another limitation refers to the inexistence of national and international studies on specific cut-points in the evaluation of adherence using the QRSD, which makes comparisons with other investigations impossible.

## Conclusions

Assessment of knowledge, barriers and attitudes towards dietary sodium among patients with HF in two different scenarios - outpatient and emergency services - allowed the determination of cut-points for satisfactory adherence to dietary sodium reduction. Countries with similar cultures may use this cut-point, as other researchers could also use it as reference in further studies.

We suggest this cut-point to identify facilitators and barriers related to reduction of dietary sodium intake in HF patients in Brazil, and be used to guide strategies, seeking better results.
